# Increasing levels of wild-type CREB up-regulates several *activity-regulated inhibitor of death* (AID) genes and promotes neuronal survival

**DOI:** 10.1186/1471-2202-13-48

**Published:** 2012-05-18

**Authors:** Yan-Wei Tan, Tina Hoffmann, Hilmar Bading

**Affiliations:** 1 Department of Neurobiology, Interdisciplinary Center for Neurosciences, Im Neuenheimer Feld 364, Heidelberg, 69120, Germany; 2 Kavli Institute for Systems Neuroscience, Centre for the Biology of Memory, Norwegian University of Science and Technology, Trondheim, 7489, Norway

**Keywords:** CREB, Neuroprotection, Hippocampal neurons, Nuclear calcium, CaMKIV, Gene expression, Viral gene delivery, Recombinant adeno-associated virus

## Abstract

**Background:**

CREB (cAMP-response element binding protein) is the prototypical signal-regulated transcription factor. In neurons, it is the target of the synaptic activity-induced nuclear calcium-calcium/calmodulin dependent protein kinase (CaMK) IV signaling pathway that controls the expression of genes important for acquired neuroprotection as well as other long-lasting adaptive processes in the nervous system. The function of CREB as a transcriptional activator is controlled by its phosphorylation on serine 133, which can be catalyzed by CaMKIV and leads to the recruitment of the co-activator, CREB binding protein (CBP). Activation of CBP function by nuclear calcium-CaMKIV signaling is a second regulatory step required for CREB/CBP-mediated transcription.

**Results:**

Here we used recombinant adeno-associated virus (rAAV) to increase the levels of wild type CREB or to overexpress a mutant version of CREB (mCREB) containing a serine to alanine mutation at position amino acid 133 in mouse hippocampal neurons. Increasing the levels of CREB was sufficient to boost neuroprotective activity even under basal conditions (i.e., in the absence of stimulation of synaptic activity). In contrast, overexpression of mCREB increased cell death. The ratio of phospho(serine 133)CREB to CREB immunoreactivity in unstimulated hippocampal neurons was similar for endogenous CREB and overexpressed wild type CREB and, as expected, dramatically reduced for overexpressed mCREB. A gene expression analysis revealed that increased expression of CREB but not that of mCREB in hippocampal neurons led to elevated expression levels of *bdnf* as well as that of several members of a previously characterized set of *Activity-regulated Inhibitor of Death* (AID) genes, which include *atf3*, *btg2*, *gadd45β*, and *gadd45γ*.

**Conclusions:**

Our findings indicate that the expression levels of wild type CREB are a critical determinant of the ability of hippocampal neurons to survive harmful conditions. Increasing the levels of wild type CREB can, even without inducing synaptic activity, increase pro-survival gene expression and strengthen the neurons’ neuroprotective shield. The observed degradation of CREB protein following NMDA treatment of hippocampal neurons suggests that the known CREB shut-off associated with extrasynaptic NMDA receptor-induced excitotoxicity is followed by CREB proteolysis.

## Background

In neurons, the transcription factor CREB and its co-activator CBP are central players in synaptic activity-driven gene transcription that contributes to the process through which neurons convert signals from the environment into genomic responses required for long-lasting adaptations [1-3]. Acquired neuroprotection is a well-studied adaptive response in which neurons that have undergone periods of synaptic activity are rendered more resistant to harmful conditions [2,4-11]. This built-up of a neuroprotective shield is initiated by calcium entry though synaptic NMDA receptors and requires calcium transient invading the cell nucleus [8-12]. Nuclear calcium acting principally via nuclear CaMKIV leads to phosphorylation of CREB on its activator site serine 133, allowing CREB to form a complex with CBP [13]. The second regulatory step required for induction of CREB/CBP-mediated transcription is the activation of CBP function by nuclear calcium-CaMKIV signaling [14-16]. Transcriptome analyses have identified a set of neuroprotective genes that are induced by synaptic activity and controlled by nuclear calcium signaling [9,10]. Several members of this group of genes, which were termed *Activity-regulated Inhibitor of Death* (AID) genes, are known or putative CREB target genes [10,11]. In addition to the ability of CREB to mediate the process that leads to ‘added-on’ survival activity upon synaptic stimulation, the presence of CREB seems to be required for the health of the neurons even under conditions of basal synaptic activity. Mice that lack CREB (and its close relative cAMP response element modulator, CREM) exhibit widespread cell death in the brain [17], and the reduction of functional CREB by means of expression of inhibitors of CREB can severely compromise the well being of neuron [18-20]. Given that physiological expression levels of functional CREB appear to be required cell survival we reasoned that increasing the levels of CREB in hippocampal neurons may enhance neuroprotection even under basal conditions. Here we have tested this hypothesis and found that indeed overexpression of wild type CREB, even without inducing synaptic activity, increases the expression of several *AID* genes and renders hippocampal neurons more resistant to cell death inducing conditions. Consistent with the importance of having physiological levels of functional CREB available at promoter regions for the maintenance of cell vitality, expression of mCREB increased cell death.

## Results and discussion

### Expression and serine 133 phosphorylation of wild type and mutant CREB

**Figure 1:**
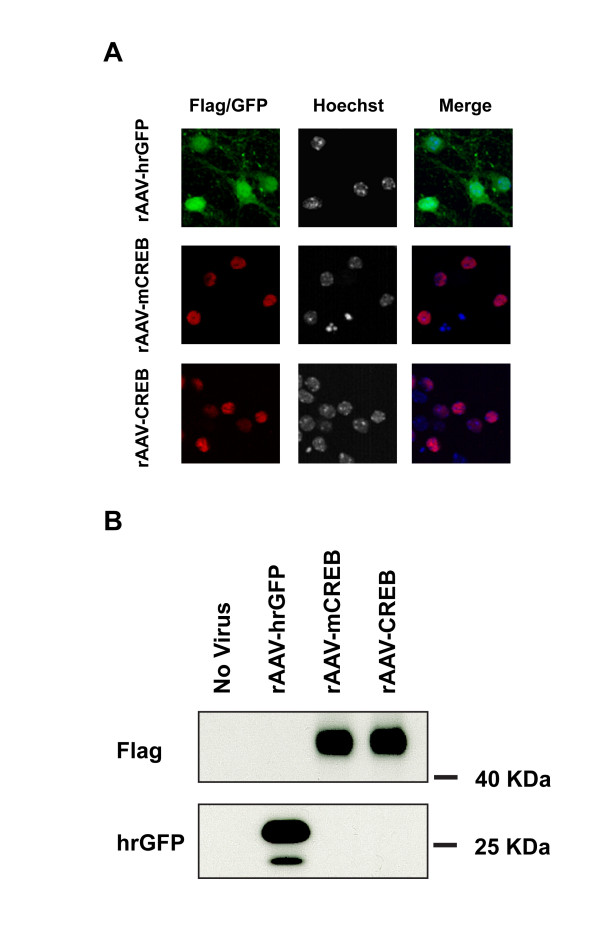
**Immunocytochemical (A) and immunoblot analyses (B) of hippocampal neurons infected with rAAVs containing expression cassettes for hrGFP, CREB or mCREB. CREB and mCREB contain triple Flag epitope tags and were detected with M2 Flag antibody; hrGFP was detected with an antibody to hrGFP.** Cell nuclei were counterstained with Hoechst 33258. Representative images are shown. Scale bar, 10 μm.

To investigate the relationship between cellular CREB levels and neuronal survival activity, we infected primary mouse hippocampal neurons with a recombinant adeno-associated virus containing an expression cassette for wild type rat CREB, a mutant version of CREB (mCREB) containing a serine to alanine mutation at position amino acid 133, or hrGFP (humanized *Renilla reniformis* green fluorescent protein). Expression of all three proteins following infection of mouse hippocampal neurons with rAAV-CREB, rAAV-mCREB, or rAAV-hrGFP was readily detectable immunocytochemically or GFP fluorescence in 80 to 95% of the viable cells (Figure 1A). The correct size proteins were also detected in immunoblots (Figure 1B; note that due to the fusion of the exogenously expressed CREB and mCREB to a triple Flag tag, the size of the exogenously expressed CREB and mCREB is slightly larger than that of the endogenous CREB).

**Figure 2:**
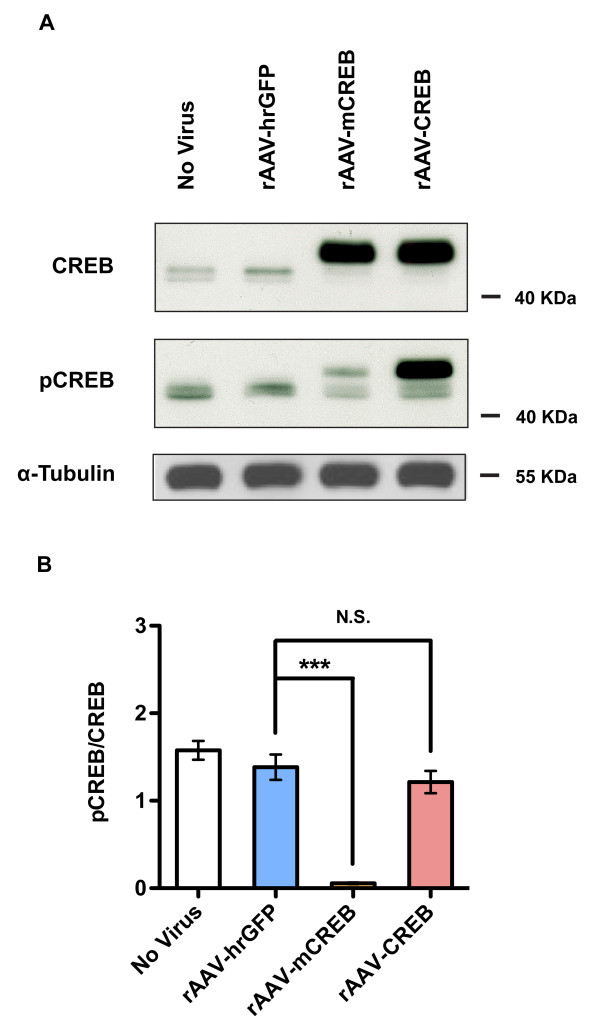
**(A) Immunoblot analysis of expression and phosphorylation on serine 133 of endogenous and overexpressed CREB in uninfected hippocampal neurons and in neurons infected with rAAV-hrGFP, rAAV-CREB and rAAV-mCREB. CREB expression and CREB phosphorylation on serine 133 (pCREB) was assessed using antibodies to CREB and antibodies specific for the serine 133 phosphorylated form of CREB, respectively.** (**B**) Quantitative analysis of the levels of CREB and CREB phosphorylated on serine 133 (pCREB). In uninfected hippocampal neurons and in neurons infected with rAAV-hrGFP, the ratio of pCREB to CREB (pCREB/CREB) was calculated for endogenous CREB; for hippocampal neurons infected with rAAV-CREB and rAAV-mCREB, the ratios were determined for overexpressed CREB and mCREB. Bars represent means ± SEM (n = 3). Statistical significance was determined by One-way ANOVA, Bonferroni post hoc test; statistically significant differences are indicated with an asterisk (***p < 0.001 versus rAAV-hrGFP; N.S., not significant).

Analysis of the phosphorylation of CREB on serine 133 in unstimulated hippocampal neurons using phospho(serine133)CREB-specific antibodies revealed very weak signals for the endogenous CREB protein in all samples analyzed (Figure 2A). We detected much stronger phospho(serine133)CREB immunoreactivity for the overexpressed CREB protein, while, as expected, low levels of phospho(serine133)CREB immunoreactivity were obtained for mCREB. The ratio of phospho(serine133)CREB to CREB immunoreactivity was similar for the endogenous CREB and the overexpressed wild type CREB, but, in contrast, was dramatically reduced for overexpressed mCREB (Figure 2B). This suggests that the strong phospho(serine133)CREB signal obtained using lysates from hippocampal neurons infected with rAAV-CREB reflects the large amount of CREB protein present in the cells, which compared to the endogenous CREB appears to contain a similar fraction of the serine 133-phosphorylated form. However, we cannot rule out the possibility that the phospho(serine133)CREB-specific antibody solution contains a small fraction of antibodies that bind to CREB even when it is not phosphorylated on serine 133. In this scenario, the phospho(serine133)CREB immunoreactivity detected in samples from rAAV-CREB infected neurons may not indicate the presence of a subset of serine133-phosphorylated CREB molecules but it is merely the result of the binding to CREB of the fraction of the antibody solution that can interact with the non-phosphorylated epitope. The observation that mCREB yielded a very weak signal with the phospho(serine133)CREB-specific antibody solution does not argue against this possibility because the mutation of serine 133 not only abolished any possible phosphorylation event at amino acid 133 but may also alter the epitope thereby reducing the binding to CREB of those antibodies (within the solution of the phospho(serine133)CREB-specific antibodies) that recognize CREB not phosphorylated on serine 133. The antibody used to detect total CREB levels was generated using a recombinant N-terminal fragment of CREB as an antigen.

### Elevated CREB level protects against apoptosis and excitotoxicity

**Figure 3:**
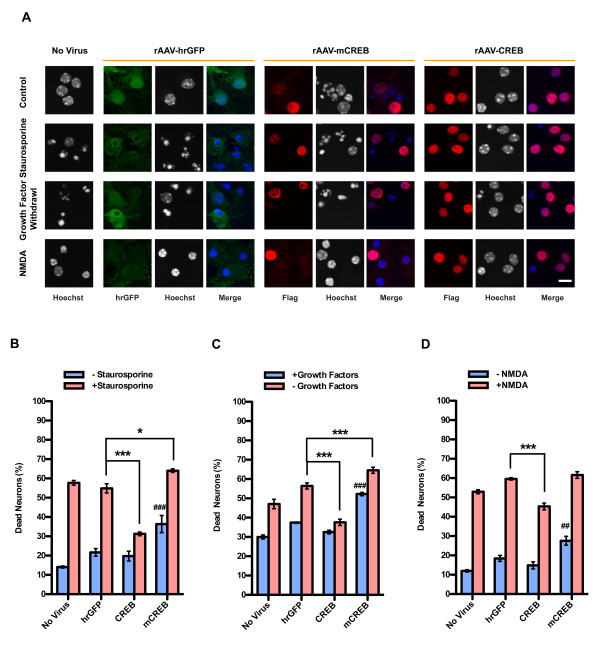
**Increasing levels of CREB protects against apoptosis and excitotoxicity. (A) Analysis of cell death in uninfected hippocampal neurons and in hippocampal neurons infected with rAAVs-hrGFP, rAAV-CREB or rAAV-mCREB.** CREB and mCREB were detected with the M2 Flag antibody; hrGFP was detected with an antibody to hrGFP. Cell death was determined by analyzing Hoechst stained nuclei with shrunken cell body and large round chromatin clumps. For mCREB expression we obtained very weak Flag immunoreactivity in the death-inducing conditions, which is most likely due to compromised cell health following mCREB expression. Representative images are shown. Scale bar, 10 μm. Quantitative analysis of cell death induced by 10 nM staurosporine (**B**), growth factor withdrawal (**C**), or bath application of 20 μM NMDA (**D**). Bars represent means ± SEM (n = 4). Statistical significance was determined by Two-way ANOVA, Bonferroni post hoc test; statistically significant differences are indicated (***p < 0.001, **p < 0.01, *p < 0.05 compared to rAAV-hrGFP infected neurons in groups induced to undergo cell death; ###p < 0.001, ##p < 0.01 compared to rAAV-hrGFP infected neurons in untreated groups).

In order to investigate a possible neuroprotective effect of elevating intracellular CREB levels, hippocampal neurons were infected with rAAV-mCREB, rAAV-CREB, rAAV-hrGFP on day *in vitro* (DIV) 4. Six days post-infection, the neurons were challenged using three different types of cell death-inducing conditions. We treated neurons with a low concentration of staurosporine and growth factor withdrawal to induce apoptosis, and bath application of 20 μM NMDA to trigger excitotoxicity [10,11]. We found that in all three types of cell death assays, the stimulus-induced increase in the percentage of dead neurons was smaller in hippocampal cultures infected with rAAV-CREB compared to the uninfected or rAAV-hrGFP infected control (Figure 3A-D). This indicates that increasing the levels of CREB in hippocampal neurons renders the cells more resistant to apoptosis and excitotoxicity. In contrast, overexpression of mCREB failed to provide neuroprotection and, instead, increased the basal death rates (Figure 3A-D). Because mCREB can bind to the CRE [21], it competes with endogenous CREB for docking sites within gene regulatory regions. This may explain the cell death-promoting activity of mCREB, consistent with the hypothesis that a reduction below physiological levels of functional CREB available at target gene promoters renders neurons more susceptible for harmful conditions.

### NMDA-induced CREB phosphorylation, CREB shut-off, and CREB degradation

**Figure 4:**
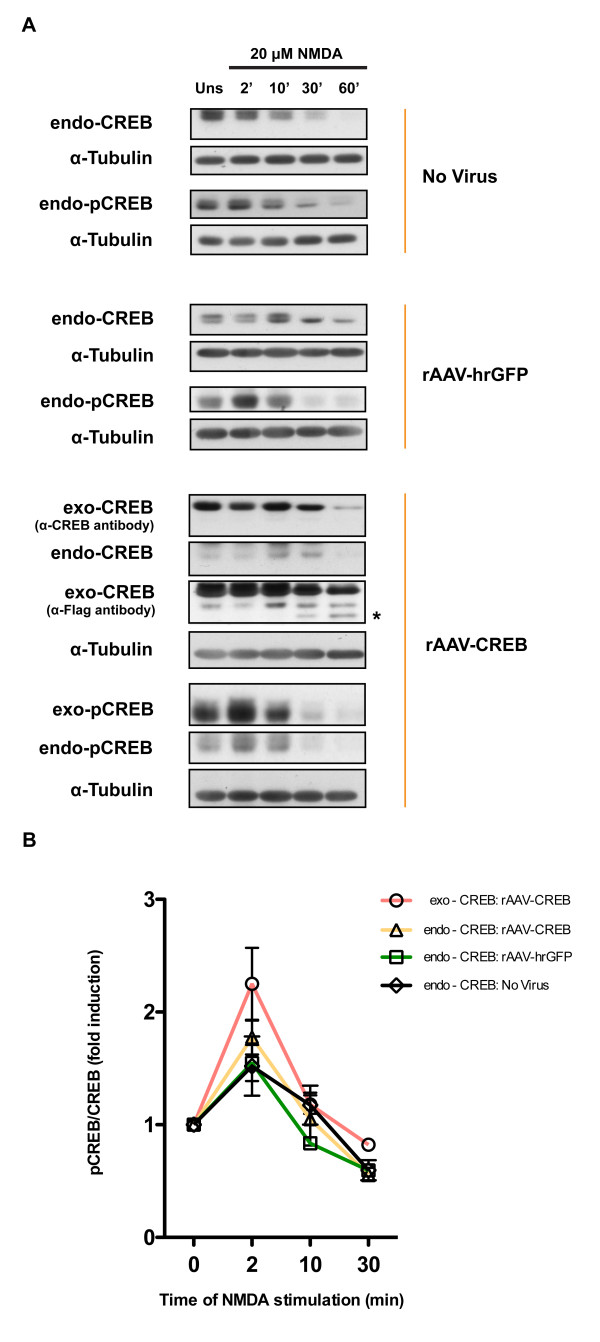
(**A) Immunoblot analysis of expression and phosphorylation on serine 133 of endogenous CREB (endo-CREB; endo-pCREB) and overexpressed CREB (exo-CREB; exo-pCREB) in uninfected hippocampal neurons and in neurons infected with rAAV-hrGFP or rAAV-CREB at the indicated times after treatment with 20 μM NMDA.** Expression of endogenous and overexpressed CREB was analyzed with an antibody to CREB; overexpressed CREB was also detected using an anti-Flag antibody. CREB phosphorylation on serine 133 (pCREB) was assessed using antibodies specific for the serine 133 phosphorylated form of CREB. CREB degradation products are indicated by an asterisk. (**B**) Quantitative analysis of the pCREB/CREB ratios of the immunoblot analyses shown in (**A**). Means ± SEM are given (n = 4).

Increasing the levels of CREB expression in hippocampal neurons did not change the dynamics of CREB phosphorylation on serine 133 after exposure to 20 μM NMDA. We found that the ratio of phospho(serine133)CREB immunoreactivity (pCREB) to CREB immunoreactivity increased very transiently after stimulation in uninfected hippocampal neurons and in neurons infected with rAAV-CREB and rAAV-hrGFP (Figure 4A, B). Peak levels of the pCREB/CREB immunoreactivities for both endogenously and exogenously expressed CREB were observed at 2 min after stimulation, which is followed by a rapid shut-off of CREB phosphorylation on serine 133 (Figure 4A, B). Because we observed significant degradation of CREB at 60 min after NMDA treatment (Figure 4A), a meaningful ratio of pCREB to CREB immunoreactivities cannot be determined for this time point. The degradation of CREB protein after induction of excitotoxicity, which is already detectable 30 min after exposure to NMDA, appears to start within the amino-terminus of CREB because degradation products of exogenously expressed CREB, Flag-tagged at the carboxyl-terminus, are detected with antibodies to Flag but not with antibodies to CREB that recognize an amino-terminal epitope (Figure 4A, CREB degradation products are indicated by an asterisk). It is tempting to speculate that CREB degradation, which may be catalyzed by the calcium dependent protease, calpain, is causally involved in the cell death process. Thus, excitotoxicity triggered by the stimulation of extrasynaptic NMDA receptors leads in a first phase to a rapid CREB shut-off (i.e., dephosphorylation of phospho(serine133) of CREB) [7], which is followed by the degradation of CREB protein. Overexpression of CREB delays the excitotoxicity-associated depletion of CREB from the neurons, which likely contributes to neuroprotection.

### CREB regulation of survival gene expression

**Figure 5:**
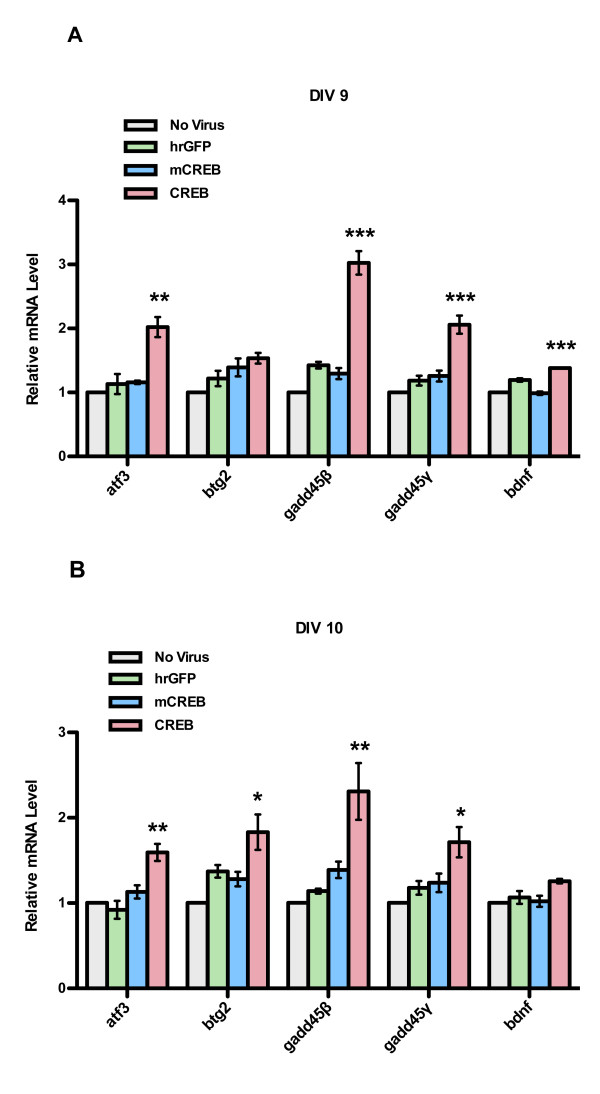
**QRT-PCR analysis of*atf3*,*btg2*,*gadd45β*, and*gadd45γ*and*bdnf*expression in uninfected hippocampal neurons and in neurons infected with rAAV-hrGFP, rAAV-CREB and rAAV-mCREB.** Gene expression analyses were done on DIV 9 (**A**) and DIV 10 (**B**), which correspond to five days and six days, respectively, post-infection. Bars represent SEM (n = 3). Statistical significance was determined by One-way ANOVA, Bonferroni post hoc test, statistically significant differences are indicated with an asterisk (***p < 0.001, **p < 0.01, *p < 0.05).

In a previous study we have identified a set of synaptic activity- and nuclear calcium-regulated neuroprotective genes, which were termed *Activity-regulated Inhibitor of Death* (AID) genes [10] Several AID genes are known or putative CREB target genes [10,11]. We therefore investigated the possibility that the observed neuroprotection following infection with rAAV-CREB is associated with an increase in *AID* gene expression. Compared to uninfected hippocampal neurons and neurons infected with rAAV-hrGFP or rAAV-mCREB, we detected a 2- to 3- fold increase in the expression of the *AID* genes, *atf3btg2gadd45β*, and *gadd45γ* after 5 and/or 6 days post infection (i.e., DIV 9 or DIV 10) with rAAV-CREB (Figure 5A, B). We also observed a slight but significant increase in the *bdnf* expression 5 days after infection with rAAV-CREB (Figure 5A). These findings suggest that neuroprotection afforded by elevated CREB levels may be the result of increased expression of neuroprotective genes.

## Conclusions

Our results indicate that increasing cellular CREB levels can enhance the ability of hippocampal neurons to survive neurotoxic stimuli. This form of CREB-mediated neuroprotection is being built up under conditions of basal neuronal activity and is associated with an increase of several survival promoting genes.

## Methods

### Hippocampal cell culture

Hippocampal neurons from newborn C57Black6 mice were prepared as described previously [9,22,23]. Neurons were plated onto either 12 mm glass coverslips or 35 mm dishes at a density between 400 and 600 cells per mm^2^ and cultured in Neurobasal media (Invitrogen, Gaithersburg, MD, USA) containing 1% rat serum and B27 (Invitrogen), and penicillin and streptomycin (Sigma). On days *in vitro* (DIV) 3, 2.4 μM cytosine D-arabinofuranoside (Sigma) was added to each dish to prevent proliferation of non-neuronal cells. At DIV 8, medium was replaced with transfection medium (TM) [22] which consists of a salt-glucose-glycine (SGG) solution and minimum Eagle’s medium (9:1; v/v) with sodium selenite 10 μg/ml, insulin 15 μg/ml, transferring 8.25 μg/ml, and penicillin-streptomycin 0.5%. SGG includes (in mM): NaCl 114, NaHCO_3_ 26, KCl 6.3, MgCl_2_ 1, CaCl_2_ 2, Hepes 10, glycine 1, glucose 30, sodium pyruvate 0.5, Phenol Red 0.2%. All the experiments were done after a culturing period of 10 to 13 days during which hippocampal neurons develop a rich network of processes, express function NMDA-type and AMPA/kainite-type glutamate receptors, and form synaptic contacts [7,24].

### Recombinant adeno-associated viruses

The vectors used to construct and package rAAVs have been described previously [9,10,25]. The recombinant virus for the expression of the humanized Renilla reniformis green fluorescent (hrGFP) was generated as described in previous work [9]. A recombinant adeno-associated virus vector containing 1 kbp cytomegalovirus enhancer (CMV)/chicken β actin hybrid promoter (CBA) was used to express CREB and mCREB. Both rAAV-CREB and rAAV-mCREB contain triple Flag epitope tag. All the vectors were generated by standard molecular biology techniques and verified by sequencing. Viral particles were produced and purified as described previously [9,10,26,27]. For viral infection, neurons were infected with 10^11^ rAAV particles/μL at DIV 4. Infection efficiencies were determined immunocytochemically at DIV 9 or 10 by using antibodies to the Flag tag, or by analyzing the fluorescence of hrGFP; they ranged from 80%-95% of the viable neurons.

### Immunocytochemistry

Mouse hippocampal neurons were fixed with 4% paraformaldehyde for 15 min and permeabilized with 0.3% Triton X 100 in PBS for 10 min. PFA was blocked by 10 min incubation in 1.25 M Glycine. After washing in PBS, the cells were blocked with blocking buffer (2% BSA, 10% normal goat serum in PBS) at room temperature for 1 h. The cells were exposed to primary antibody (anti-Flag M2 Flag antibody, Sigma, MO, US) in dilution buffer (2% BSA, 0.1% Triton X 100 in PBS) at 4°C overnight, washed in PBST (0.1% Tween 20 in PBS) and exposed to secondary antibodies in dilution buffer. After washing with PBST, the cells were incubated with Hoechst 33258 for 5 min, washed in PBS and mounted with mowiol and glycerol mounting medium, and dried at room temperature overnight. The cells were examined by fluorescent microscopy using a Leica SP2 confocal microscope (Leica, Wetzlar, Germany).

### Immunoblot analysis

Hippocampal neurons incubated under appropriate conditions were washes with ice-cold PBS and cell lysates were prepared. The lysates were mixed with 5 × Laemmli sample buffer and boiled for 5 min. The proteins were resolved on 10% SDS-polyacrylamide gels and transferred to nitrocellulose (NC) membranes. The blots were blocked with Tris-buffered saline with Tween-20 (TBST; 20 mM Tris–HCl [PH 7.6], 0.136 M NaCl and 0.5% Tween-20 [vol./vol.]) containing 5% skim milk at room temperature for 1 h followed by incubation of primary antibody in TBST containing 5.0% BSA at 4°C overnight. Expression of CREB and phospho-CREB (pCREB) were measured by immunoblotting using the antibodies to the CREB (rabbit monoclonal antibody; Cell Signaling Technology, MA, USA) and pCREB (rabbit polyclonal antibody; Upstate, MA, USA); hrGFP expression levels were detected using antibody to the hrGFP (rabbit polyclonal antibody; Stratagene); tubulin (mouse monoclonal antibody; Sigma, MO, US) was chosen as the loading control. After washing with TBST, goat-anti-rabbit or mouse HRP conjugated IgG (Promega, WI, US) were added for 1 h at room temperature. Blots were then washes with TBST and exposed to X-ray film. The blots were quantitatively analyzed using ImageJ (http://rsb.info.nih.gov/ij/). All results are given as means ± SEM; statistical significance was determined by One-way ANOVA, Bonferroni post hoc test.

### Quantitative reverse transcriptase PCR

To determine the mRNA levels of the potential pro-survival genes regulated by CREB, QRT-PCR was performed using real-time TaqMan technology with a sequence detection system model 7300 Real Time PCR System (Applied Biosystems, Foster City, CA, USA). Total RNA was extracted using RNeasy kit (Qiagen GmbH, Germany) with additional on-column DNase I digestion during RNA purification. cDNA was generated from 1.5 μg of total RNA using High Capacity cDNA Reverse Transcription kit (Applied Biosystems). QRT-PCR was carried out using TaqMan Universal PCR Master Mix (Applied Biosystems). The following TaqMan gene expression assays were used in this study: *atf3* (Mm00476032), *bdnf* (Mm00432069), *btg2* (Mm00476162), *gadd45β* (Mm00435123), *gadd45γ* (Mm00442225), *gusb* (Mm00446953_m1). The thermal cycling conditions comprised 10 min at 95°C, and 45 cycles of 15 s for denaturation at 95°C and 60 s for annealing and extension at 60°C. The expression levels of the target mRNA were normalized to the relative ratio of the expression of *gusb* mRNA. Each QRT-PCR assay was performed three times. All results are given as means ± SEM; statistical significance was determined by One-way ANOVA, Bonferroni post hoc test.

### Assessment of cell death

The induction and analysis of NMDA induced neuronal cell death assay was done as described with slight changes [11,27,28]. Briefly, the cells were treated with 20 μM NMDA for 10 min at 37°C, washed three times with TM and incubated at 37°C for 20 h. The percentage of dead cells was determined by analyzing Hoechst 33258 stained nuclei, and the percentage of dead cells was determined. The Staurosporine induced and growth factor withdrawal induced apoptosis assay have been described previously [9-11]. Briefly, 36 h after staurosporine (10 nM) exposure, or 72 h after keeping hippocampal neurons in TM medium minus the growth and trophic factors, all in the presence of 1 μM tetrodotoxin (TTX, Tocris Bioscience), cell death was assessed by determining the percentage of hippocampal neurons with shrunken cell body and large round chromatin clumps. All the cell death assays were done at DIV 10-13, at least 20 visual fields from each coverslip (corresponding to 1500–2000 cells per coverslip) were counted. All results are given as means ± SEM; statistical significance was determined by Two-way ANOVA, Bonferroni post hoc test.

## Abbreviations

atf3: Activating transcription factor 3; btg2: B-cell translocation gene 2; gadd45β: Growth arrest and DNA-damage-inducible, beta; gadd45γ: Growth arrest and DNA-damage-inducible, gamma; bdnf: Brain-derived neurotrophic factor; CaMKIV: Calcium/calmodulin-dependent protein kinase 4; CBP: CREB binding protein; CREM: cAMP response element modulator.

## Authors’ contributions

HB conceived the study and participated in its design. YWT designed and performed the cell death assay, the immunoblot experiments, and the quantitative PCR assay. SJZ generated the rAAV constructs and viruses. TH carried out part of the gene expression analysis. HB and YWT wrote the manuscript. All authors approved the final manuscript.
